# Determinants of fluconazole resistance and the efficacy of fluconazole and milbemycin oxim combination against *Candida parapsilosis* clinical isolates from Brazil and Turkey

**DOI:** 10.3389/ffunb.2022.906681

**Published:** 2022-07-28

**Authors:** Farnaz Daneshnia, Süleyha Hilmioğlu Polat, Macit Ilkit, Erika Shor, João Nobrega de Almeida Júnior, Larissa M. Favarello, Arnaldo Lopes Colombo, Amir Arastehfar, David S. Perlin

**Affiliations:** ^1^ Center for Discovery and Innovation, Hackensack Meridian Health, Nutley, NJ, United States; ^2^ Institute for Biodiversity and Ecosystem Dynamics, University of Amsterdam, Amsterdam, Netherlands; ^3^ Department of Medical Microbiology, Ege University Faculty of Medicine, Izmir, Turkey; ^4^ Division of Mycology, Faculty of Medicine, Çukurova University, Adana, Turkey; ^5^ Hackensack Meridian School of Medicine, Nutley, NJ, United States; ^6^ Laboratorio de Micologia Medica (LIM 53), Instituto de Medicina Tropical, Universidade de São Paulo, São Paulo, Brazil; ^7^ Laboratório Central (LIM 03), Hospital das Clínicas da Faculdade de Medicina da Universidade de São Paulo, São Paulo, Brazil; ^8^ Department of Medicine, Division of Infectious Diseases, Escola Paulista de Medicina, Universidade Federal de São Paulo, São Paulo, Brazil; ^9^ Georgetown University Lombardi Comprehensive Cancer Center, Washington, DC, United States

**Keywords:** *Candida parapsilosis*, outbreak, candidemia, fluconazole resistance, fluconazole potentiation

## Abstract

Fluconazole-resistant *Candida parapsilosis* (FLZR-CP) outbreaks are a growing public health concern and have been reported in numerous countries. Patients infected with FLZR-CP isolates show fluconazole therapeutic failure and have a significantly increased mortality rate. Because fluconazole is the most widely used antifungal agent in most regions with outbreaks, it is paramount to restore its antifungal activity. Milbemycin oxim (MOX), a well-known canine endectocide, is a potent efflux pump inhibitor that significantly potentiates the activity of fluconazole against FLZR *C*. *glabrata* and *C*. *albicans.* However, the FLZ-MOX combination has not been tested against FLZR-CP isolates, nor is it known whether MOX may also potentiate the activity of echinocandins, a different class of antifungal drugs. Furthermore, the extent of involvement of efflux pumps *CDR1* and *MDR1* and ergosterol biosynthesis enzyme *ERG11* and their link with gain-of-function (GOF) mutations in their transcription regulators (*TAC1*, *MRR1*, and *UPC2*) are poorly characterized among FLZR-CP isolates. We analyzed 25 C. *parapsilosis* isolates collected from outbreaks in Turkey and Brazil by determining the expression levels of *CDR1*, *MDR1*, and *ERG11*, examining the presence of potential GOF mutations in their transcriptional regulators, and assessing the antifungal activity of FLZ-MOX and micafungin-MOX against FLZR and multidrug-resistant (MDR) *C*. *parapsilosis* isolates. *ERG11* was found to be universally induced by fluconazole in all isolates, while expression of *MDR1* was unchanged. Whereas mutations in *MRR1* and *UPC2* were not detected, *CDR1* was overexpressed in three Brazilian FLZR-CP isolates, which also carried a novel *TAC1^L518F^* mutation. Of these three isolates, one showed increased basal expression of *CDR1*, while the other two overexpressed *CDR1* only in the presence of fluconazole. Interestingly, MOX showed promising antifungal activity against FLZR isolates, reducing the FLZ MIC 8- to 32-fold. However, the MOX and micafungin combination did not exert activity against an MDR *C*. *parapsilosis* isolate. Collectively, our study documents that the mechanisms underpinning FLZR are region specific, where *ERG11* mutations were the sole mechanism of FLZR in Turkish FLZR-CP isolates, while simultaneous overexpression of *CDR1* was observed in some Brazilian counterparts. Moreover, MOX and fluconazole showed potent synergistic activity, while the MOX-micafungin combination showed no synergy.

## Introduction

As commensals inhabiting various mucosal surfaces in humans, species belonging to the genus *Candida* are responsible for approximately 1.5 billion superficial and almost 1.5 million systemic infections annually worldwide and therefore pose a serious threat to public health (Brown et al., 2012). Although the advent of antifungal drugs has improved the clinical outcomes of afflicted patients, a shift in epidemiology and the emergence of drug-resistant fungi, such as *C*. *glabrata* and *C*. *auris*, have challenged the efficacy of the limited number of antifungal drugs available to treat candidiasis (Ahangarkani et al., 2020; Arastehfar et al., 2020a). Recently, severe clonal outbreaks due to fluconazole-resistant (FLZR) *C*. *parapsilosis* have been reported in numerous countries (Choi et al., 2018; Thomaz et al., 2018; Singh et al., 2019; Martini et al., 2020; Arastehfar et al., 2020a; Arastehfar et al., 2020b; Arastehfar et al., 2021; Corzo-Leon et al., 2021; Fekkar et al., 2021), which is a matter of serious concern because azole-naïve patients infected with FLZR *C*. *parapsilosis* show clinical failure to azoles, complicating the treatment, prolonging the duration of hospitalization, and increasing hospital-related costs. Most importantly, analyses of cohorts from Turkey and Brazil have revealed that patients infected with FLZR *C*. *parapsilosis* isolates have a significantly higher rate of mortality than patients infected with susceptible isolates (Arastehfar et al., 2020b; Arastehfar et al., 2021; Thomaz et al., 2021). Alarmingly, FLZR *C*. *parapsilosis* isolates continue to expand in clinical settings and replace their susceptible counterparts even when strict infection control strategies are applied (Thomaz et al., 2021). Thomaz et al. (2021) showed that FLZR *C*. *parapsilosis* from the hands of healthcare workers and the hospital environment share 100% genetic similarity with isolates obtained from the bloodstream of afflicted patients. Moreover, the recent emergence of genetically related multidrug-resistant *C*. *parapsilosis* exhibiting resistance to both frontline-used antifungal drugs, azoles and echinocandins, presents a new clinical challenge (Arastehfar et al., 2020c). Therefore, the severity of drug-resistant *C*. *parapsilosis* clonal outbreaks can be even higher in countries where this species is considered the second leading cause of candidemia, including South American (Nucci et al., 2013), South African (Govender et al., 2016), some European (Siopi et al., 2020), Mediterranean (Arikan-Akdagli et al., 2019), and South/East Asian countries (Kakeya et al., 2019; Guo et al., 2021).

Azole resistance in *C*. *parapsilosis* is mainly driven by drug target mutations in the *ERG11* gene, including Y132F, K143R, Y132F+K143R, Y132F+G307A, and G458S (Grossman et al., 2015; Choi et al., 2018; Thomaz et al., 2018; Arastehfar et al., 2020b; Arastehfar et al., 2020c; Arastehfar et al., 2021; Corzo-Leon et al., 2021). Of note, FLZR *C*. *parapsilosis* isolates collected from different countries show either a narrow range of mutations in *ERG11*, such as Y132F in Brazil (Thomaz et al., 2018, 2021), South Korea (Choi et al., 2018), France (Fekkar et al., 2021), and Mexico (Corzo-Leon et al., 2021), or a high degree of variability of mutations, such as all of the aforementioned mutations found in Turkish isolates (Arastehfar et al., 2020a; Arastehfar et al., 2021). Although poorly studied in *C*. *parapsilosis*, other mechanisms underpinning azole resistance have been well characterized in *C*. *albicans*, including overexpression of the drug target *ERG11* as well as of efflux pumps, namely, ATP-binding cassette (ABC) transporters (such as *CDR1*) and the major facilitator superfamily (such as *MDR1*) (Arastehfar et al., 2020b). The overexpression of *CDR1*, *MDR1*, and *ERG11* typically involves gain-of-function (GOF) mutations in their transcriptional regulators, i.e., *TAC1* (*PDR1* ortholog in *C*. *glabrata*), *MRR1*, and *UPC2*, respectively (Arastehfar et al., 2020a). Interestingly, analysis of a comprehensive collection of clinical FLZR *C*. *albicans* isolates has shown that the overexpression of *CDR1* and *ERG11* and occasionally *MDR1* almost always occur in combination with *ERG11* mutations (Flowers et al., 2015). Moreover, it has been suggested that the overexpression of efflux pumps due to such GOF mutations is a virulence factor in *C*. *glabrata* (Ferrari et al., 2009).

Azoles are one of the most widely used antifungals, especially in developing countries (Arastehfar et al., 2019), and are most severely affected by FLZR *C*. *parapsilosis*. Given the extensive involvement of ABC transporters in azole resistance, some *in vitro* studies have taken advantage of ABC transporter inhibitors in combination with fluconazole, which have shown promising efficacy when tested *in vitro* and *in vivo* (Silva et al., 2013; Iyer et al., 2020). For instance, oxindole (Iyer et al., 2020) and milbemycin derivatives (Silva et al., 2013) have shown prominent efficacy against *C*. *auris* (Iyer et al., 2020) and *C*. *albicans* and *C*. *glabrata* (Silva et al., 2013), respectively. Milbemycin derivatives are particularly promising because they exert a broader activity and have a long half-life and low cytotoxicity (Silva et al., 2013). Moreover, the function of milbemycin extends beyond the inhibition of ABC transporters, as is an approved endectocide in canines, and it exerts fungicidal activity on its own through generation of reactive oxygen species (ROS) (Silva et al., 2013). Expectedly, milbemycin derivatives potentiated fluconazole efficacy when tested in mice infected with FLZR *C*. *glabrata* and *C*. *albicans* isolates, resulting in fungal burdens decreasing to the same levels as those infected with susceptible counterparts (Silva et al., 2013). Interestingly, milbemycin derivatives were also found to have a favorable safety profile, tolerability, and efficacy when tested in humans, although with a limited number of patients (Cotreau et al., 2003). However, the efficacy of milbemycin against FLZR *C*. *parapsilosis* has not been investigated.

Herein, we analyzed a comprehensive collection of FLZR and FLZS *C*. *parapsilosis* isolates obtained from outbreaks in Turkey and Brazil to investigate (a) the involvement of efflux pumps and *ERG11* overexpression in azole resistance, (b) the identification of GOF mutations potentially driving this overexpression, and (c) the efficacy of milbemycin in combination with fluconazole against FLZR *C. parapsilosis* isolates.

## Materials and methods

### *Candida parapsilosis* strain collection

This study included 26 *C. parapsilsis* isolates collected from outbreaks in Turkish and Brazilian hospitals, including 21 FLZR, 4 fluconazole susceptible (FLZS), and one MDR isolate resistant to both fluconazole and echinocandins (**Table 1**). FLZR and FLZS isolates from both centers were included. Each single isolate represents a single patient. The single MDR *C*. *parapsilosis* was included to explore the efficacy of micafungin in combination with milbemycin oxim (MOX).

**Table 1:** List of *C. parapsilosis* isolates included in this study.

Strain #	FLZ	VRZ	MICA	ANI	Erg11	Fks1	Isolation year	Isolation source
***C. parapsilosis* isolates recovered from Turkish cohort**								
**T1**	0.5	0.03	1	1	WT-S	WT	2019	Blood
**T2**	0.25	0.03	0.5	1	WT-S	WT	2019	Blood
**T3**	4	0.25	0.5	1	Y132F	WT	2019	Blood
**T4**	32	0.5	1	1	Y132F	WT	2019	Blood
**T5**	4	0.06	0.5	1	Y132F	WT	2019	Blood
**T6**	8	0.125	0.5	0.5	Y132F	WT	2019	Blood
**T7**	≥8	0.25	1	2	Y132F	WT	2020	Blood
**T8**	16	0.125	1	2	Y132F	WT	2020	Blood
**T9**	32	0.25	1	2	Y132F	WT	2020	Blood
**T10**	>64	1	1	1	Y132F+G307A	WT	2019	Blood
**T11**	≥64	1	1	1	Y132F+G307A	WT	2020	Blood
**T12**	≥64	1	1	2	Y132F+G307A	WT	2020	Blood
**T13**	32	1	1	1	G458S	WT	2019	Blood
**T14**	>64	2	1	1	G458S	WT	2019	Blood
**T15**	≥64	1	0.25	0.5	G458S	WT	2020	Blood
**T16**	≥64	1	1	2	G458S	WT	2020	Blood
**T17**	≥8	0.5	≥8	4	Y132F+K143R	R658G	2020	Blood
***C. parapsilosis* isolates recovered from Brazilian cohort**								
**B1**	≥64	0.125	1	2	Y132F	WT	2020	Blood
**B2**	≥64	0.25	1	2	Y132F	WT	2019	Blood
**B3**	≥64	0.25	0.5	1	Y132F	WT	2019	Blood
**B4**	≥8	0.25	1	2	Y132F	WT	2020	Blood
**B5**	≥8	0.25	0.5	1	Y132F	WT	2020	Blood
**B6**	≥16	0.25	1	2	Y132F	WT	2020	CVC
**B7**	16	0.125	0.5	1	Y132F	WT	2020	Blood
**B8**	2	0.06	1	2	WT-S	WT	2020	Blood
**B9**	2	0.06	1	2	WT-S	WT	2020	Blood

FLZ, Fluconazole; VRZ, Voriconazole; MICA, Micafungin; ANI, Anidulafungin; CVC, Central venous catheter; WT-S, Wild-type susceptible.

### RNA extraction and gene expression analysis

*Candida parapsilosis* cultures grown overnight (150 rpm and 37°C) were washed with PBS once, and after adjusting the cultures to OD_600_ 0.5, the cell suspensions were inoculated in fresh YPD and incubated for another 6 hours (250 rpm and 37°C). Subsequently, *C*. *parapsilosis* cells were washed twice with PBS, incubated in RPMI 1640 containing one dilution below the minimum inhibitory concentration (MIC) of fluconazole (10^5^ cells/ml), incubated at 37°C and 250 rpm for 90 minutes, collected (13,000 rpm for 5 minutes) and stored at –80°C. RNA samples were extracted using a home-brew method described elsewhere (Pekmezovic et al., 2021) and were subjected to DNase treatment (QIAGEN) per the manufacturer’s suggestion. DNase-treated RNA samples were further purified using an RNeasy mini-Kit (QIAGEN) per the manufacturer’s suggestion.

qPCR was performed using the primers listed in **Supplementary Table 1** designed for the current study. Only primers with high levels of reproducibility (≥99%) and efficiency (≥92) were used in our final qPCR assays. qPCR was performed with a One-Step TB Green PrimeScript RT-PCR Kit II (Perfect Real Time, TaKaRa, Shiga, Japan) using the universal program mentioned in **Supplementary Table 1**. qPCRs containing 40 ng of RNA samples, 0.4 µM of primers, 0.8 µL of enzyme (combination of both reverse transcriptase and *Taq* polymerase), and 10 µL of buffer in a final volume of 20 µL were subjected to an Mx3005P qPCR System (Agilent Technologies, Santa Clara, USA).

Experiments were carried out in two biological and at least two technical replicates, and gene expression data were normalized against *ACT1* gene (**Supplementary Table 1**). Fold changes were determined using normalized data of *C*. *parapsilosis* cells treated with fluconazole relative to untreated initial inoculums of each sample using 2^-ΔΔCT^ as described previously (Livak and Schmittgen, 2001). Overexpression was defined as a fold change ≥2 relative to the untreated cells. Basal expression values for each untreated samples were calculated using the following formula: 2^-ΔCt^, where ΔCt refers to the Ct gene of target-Ct *ACT1*. The Ct values of target and reference genes of each replicate were normalized against the average Ct values of target and reference genes from untreated samples. Microsoft Excel was used for gene expression analysis.

### Sequencing

*TAC1*, *UPC2*, and *MRR1* were amplified by PCR and sequenced using conditions described previously (Arastehfar et al., 2020b). Contigs were assembled using SeqMan Pro (DNASTAR, Madison, WI, USA), and fully assembled and curated sequences were aligned to the reference sequences for *TAC1* (HE605204), *MRR1* (HE605205), and *UPC2* (HE605206).

### Antifungal susceptibility and checkerboard testing

Antifungal susceptibility testing used the broth microdilution of the CLSI M27-A3 protocol, which included fluconazole, micafungin, and MOX (all from Sigma-Aldrich, St. Louis, MO, USA). Plates were incubated at 37°C for 24 hours, and the MIC50 data (50% growth inhibition compared to control without drug for each given isolate) were determined by a Tecan^®^ Infinite 200 Pro microplate reader (Männedorf, Switzerland). Fluconazole MICs were reported per established clinical breakpoints as reported (Pfaller and Diekema, 2012), where a given isolate with an MIC ≥8µg/L was considered as being fluconazole resistant.

The checkerboard assay included either MOX-fluconazole or MOX-micafungin, and the MIC50 was determined as described above. Fractional inhibitory concentrations (FICs) were determined using the following formula: ΣFIC = FICA + FICB = (CFLZ/MICFLZ) + (CMOX/MICMOX), where MICFLZ and MICMOX refer to the MIC of each drug alone, while CFLZ and CMOX refer to the combination MIC of fluconazole and MOX. FIC values ≤0.5, >0.5 to ≤1, and >1 to <4 were considered synergistic, additive, and indifferent, respectively (Silva et al., 2013).

## Results and discussion

**Figure 1:**
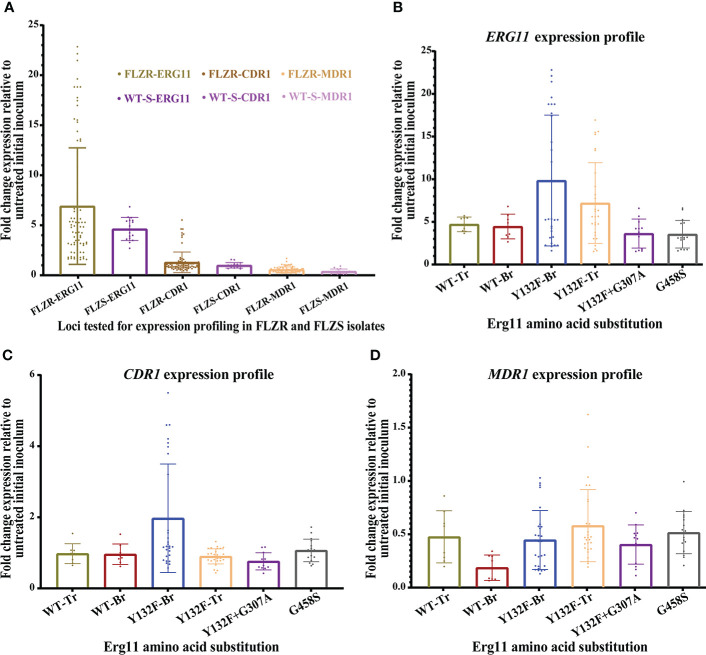
**(A)** Analysis of the expression profile of *ERG11*, *CDR1*, and *MDR1* among 25 C *parapsilosis* isolates collected from Brazilian and Turkish outbreaks. Isolates grown in logarithmic phase were treated with one dilution below the minimum inhibitory concentration of fluconazole for 90 minutes and incubated at 37°C and 250 rpm, and treated cells were subjected to RNA extraction, followed by RNase treatment and further purification of treated RNA samples, followed by qPCR. The expression data of treated cells were normalized against untreated cells, and Actin1 was used as our reference gene. The expression fold change was calculated using 2^-ΔΔCt^. **(B)** *ERG11* showed the highest level of induction irrespective of the azole susceptibility profile. **(C)** Overexpression of *CDR1* after fluconazole exposure was noted only for two Brazilian isolates. **(D)** None of the isolates overexpressed MDR1.

In this study, we used a comprehensive collection of *C*. *parapsilosis* isolates collected during outbreaks in Brazil and Turkey. *ERG11* sequencing showed that all Turkish FLZR isolates carried Y132F (*n*=7), Y132F+G307A (*n*=3), and G458S (*n*= 4), while the Brazilian counterparts only harbored Y132F (*n*=7) (**Table 1**). To determine whether changes in *ERG11*, *CDR1*, and *MDR1* expression were also involved in *C*. *parapsilosis* FLZR, the isolates were exposed to fluconazole for 90 minutes, and the expression profile of the aforementioned genes was normalized against the initial inoculum not exposed to fluconazole. Interestingly, *ERG11* showed the highest degree of induction by fluconazole regardless of susceptibility profile, *ERG11* mutation type, and country of origin (**Figures 1A, B**; **Table 2**). Moreover, the basal expression level of *ERG11* was highest in Turkish FLZR isolates carrying G458S (*P*< 0.05) but unchanged in the rest of the strains (**Supplementary Figure 1A**), raising the question about the mechanism that may confer this difference. It is possible that this mutation impairs Erg11 enzyme catalytic activity and that higher basal expression could be a strategy to compensate for the lower level of ergosterol. Because overexpression of *ERG11* is associated with a hyperactive Upc2, we sequenced this gene in all isolates, but none of the isolates harbored any mutations in *UPC2*. Therefore, *ERG11* induction is likely a universal response employed by *C*. *parapsilosis* to effectively counteract azole activity.

**Table 2:** The characteristics of *Candida parapsilosis* isolates collected from Turkish (shown by T) and Brazilian isolates.

Strain #	Fluconazole(µg/ml)	Voriconazole (µg/ml)	Erg11	*CDR1* expression	*ERG11* expression	*MDR1* expression	Tac1	Upc2	Mrr1
**T1**	0.5	0.03	WT-S	1.10 ± 0.26	5.47 ± 0.15	0.68 ± 0.12	WT	WT	WT
**T2**	0.25	0.03	WT-S	0.77 ± 0.18	3.90 ± 0.22	0.26 ± 0.05	WT	WT	ND
**T3**	4	0.25	Y132F	0.88 ± 0.18	1.77 ± 0.30	0.45 ± 0.08	WT	WT	ND
**T4**	32	0.5	Y132F	0.49 ± 0.09	13.32 ± 2.96	0.23 ± 0.03	WT	WT	ND
**T5**	4	0.06	Y132F	0.96 ± 0.15	5.99 ± 0.45	0.42 ± 0.016	WT	WT	ND
**T6**	8	0.125	Y132F	1.11 ± 0.11	6.92 ± 1.22	0.46 ± 0.08	WT	WT	ND
**T7**	≥8	0.25	Y132F	0.88 ± 0.17	3.29 ± 0.34	0.74 ± 0.17	WT	WT	ND
**T8**	16	0.125	Y132F	0.88 ± 0.07	14.15 ± 2.68	0.49 ± 0.21	WT	WT	ND
**T9**	32	0.25	Y132F	0.82 ± 0.03	4.74 ± 0.09	1.23 ± 0.3	WT	WT	WT
**T10**	>64	1	Y132F+G307A	0.64 ± 0.12	1.62 ± 0.10	0.51 ± 0.12	WT	WT	ND
**T11**	≥64	1	Y132F+G307A	0.53 ± 0.09	3.84 ± 0.44	0.50 ± 0.05	WT	WT	ND
**T12**	≥64	1	Y132F+G307A	1 ± 0.15	5.35 ± 0.98	0.17 ± 0.04	WT	WT	ND
**T13**	32	1	G458S	0.72 ± 0.08	1.69 ± 0.06	0.59 ± 0.12	WT	WT	ND
**T14**	>64	2	G458S	1.31 ± 0.26	5.66 ± 0.97	0.46 ± 0.04	WT	WT	ND
**T15**	≥64	1	G458S	1.11 ± 0.29	3.10 ± 0.26	0.70 ± 0.19	WT	WT	ND
**T16**	≥64	1	G458S	0.97 ± 0.31	3.63 ± 1.05	0.28 ± 0.05	WT	WT	ND
**B1**	≥64	0.125	Y132F	0.70 ± 0.05	19.62 ± 2.29	0.46 ± 0.12	L877P	WT	ND
**B2**	≥64	0.25	Y132F	1.26 ± 0.18	18.31 ± 2.93	0.59 ± 0.11	L877P	WT	ND
**B3**	≥64	0.25	Y132F	1.21 ± 0.25	2.07 ± 0.31	0.97 ± 0.03	L877P	WT	WT
**B4**	≥8	0.25	Y132F	1.14 ± 0.2	16.29 ± 4.55	0.27 ± 0.04	L877P	WT	ND
**B5**	**≥8**	0.25	**Y132F**	**3.29 ± 0.97**	3.41 ± 1.42	0.15 ± 0.01	**L518F**	WT	ND
**B6**	**≥16**	0.25	**Y132F**	**4.56 ± 0.63**	5.09 ± 0.39	0.45 ± 0.03	**L518F**	WT	ND
**B7**	**16**	0.25	**Y132F**	**0.79 ± 0.21**	3.97 ± 1.03	0.18 ± 0.01	**L518F**	WT	ND
**B8**	2	0.06	WT-S	0.79 ± 0.15	5.66 ± 0.83	0.07 ± 0.008	WT	WT	WT
**B9**	2	0.06	WT-S	1.05 ± 0.34	3.20 ± 0.40	0.29 ± 0.03	L877P	WT	ND

ND, Not determined.

Isolates with upregulated CDR1 and carrying TAC1 mutation potentially involved in CDR1 upregulation are boldfaced and underlined.

The expression profile values of the genes studied are based on the average ± standard deviation.

Next, we assessed the expression of *CDR1*. Unlike *ERG11*, only two Brazilian isolates showed *CDR1* induction upon fluconazole exposure (**Figure 1C**; **Table 2**). Since this overexpression only occurred in FLZR isolates, we suspected that *TAC1* may harbor a specific GOF mutation driving the overexpression of *CDR1*. Indeed, while Turkish *C*. *parapsilosis* isolates were all WT for *TAC1*, we found two mutations among Brazilian counterparts, namely, L877P and L518F, the latter occurring exclusively in the three FLZR isolates. Of these three isolates, two overexpressed *CDR1* after fluconazole induction, while the other isolate had significantly increased basal expression of *CDR1* (**Table 2**, **Supplementary Figure 1B**). Therefore, strains belonging to specific lineages may have acquired additional changes to keep *TAC1* in check, and overexpression of *TAC1* followed by *CDR1* may occur only in the presence of azoles. Such adaptation is in line with the observations that *Candida lusitaniae* isolates carrying hyperactive *MRR1* frequently acquire secondary mutations to either completely abolish *MRR1* activity or decrease the expression of *MRR1* to adapt to the dynamic environment of the host (Demers et al., 2021).

Consistent with our findings, both azole-susceptible and azole-resistant *C*. *parapsilosis* isolates recovered from South Korean hospitals harbored L877P in *TAC1* (Choi et al., 2018), suggesting that L877P may represent a polymorphism rather than a GOF mutation, while the exclusive occurrence of the novel GOF mutation, L518F, among FLZR isolates combined with the overexpression of *CDR1* reinforces the possibility that this mutation could result in hyperactivity of *TAC1* and consequent *CDR1* overexpression. Interestingly, L518F mapped to the middle homology region (MHR), which is a presumed inhibitory domain of *TAC1* that suppresses the activity of the C-terminal activating domain (Nishimoto et al., 2020). Notably, the basal expression level of *CDR1* was not different among the isolates included in the current study (**Supplementary Figure 1B**). Therefore, our data support the conclusion that *CDR1* overexpression (either basal or induced) is likely a specific mechanism employed by some FLZR *C*. *parapsilosis* strains and that L518F is a potential GOF mutation candidate associated with overexpression of *CDR1*.

Although infrequent, *MDR1* has also been suggested to play a role in azole resistance among clinical isolates of *C*. *albicans* (Flowers et al., 2015; Nishimoto et al., 2020). Therefore, we examined the expression of *MDR1*, and to our surprise, not only did none of the isolates overexpress *MDR1*, but most of the isolates even downregulated *MDR1* in response to fluconazole (**Figure 1D** and **Table 2**). Among the 25 isolates tested, only two isolates (one from Brazil and one from Turkey) did not downregulate *MDR1*, and hence, we assumed that they may have some exclusive mutations in *MRR1* maintaining the expression of *MDR1*. However, *MRR1* sequencing in these two FLZR isolates and two susceptible counterparts revealed a lack of *MRR1* mutations. Moreover, unlike *CDR1* and *ERG11*, basal overexpression of *MDR1* was not observed (**Supplementary Figure 1C**). Therefore, we suspect that *MDR1* may not represent a frequent player in azole resistance in *C*. *parapsilosis* collected from Turkey and Brazil. These findings contrast those obtained from *C*. *parapsilosis* isolates from the USA (Grossman et al., 2015), South Korea (Zhang et al., 2015), and those obtained from a Chinese patient (Choi et al., 2018), where the exclusive occurrence of “potential” *MRR1* GOF mutations was reported for FLZR isolates. Of note, *MDR1* overexpression in the absence of *MRR1* mutation was also observed (Grossman et al., 2015), which further highlights the complexity of the mechanisms underlying azole resistance. Of the mutations exclusively found in *MRR1* in the clinical FLZR isolates, only G583R has been proven to be associated with *MDR1* overexpression and azole resistance (Branco et al., 2015), and the involvement of the rest in azole resistance has yet to be studied.

Although the *C*. *parapsilosis* isolates included in the current study were randomly selected, we still observed that fluconazole resistance mechanisms varied depending on the country of origin, and our gene expression data indicated that fluconazole resistance in Turkish *C*. *parapsilosis* isolates is mainly driven by the acquisition of *ERG11* mutation, while those from Brazilian hospitals concomitantly overexpressed *CDR1*, possibly due to a GOF mutation in *TAC1*. This phenomenon might be explained by the clonal expansion of *C*. *parapsilosis* isolates in clinical settings, where the azole resistance mechanisms in a specific hospital could be determined by the most adaptable and abundant genotype/lineage. This hypothesis is in line with a previous study from Brazil, where all the FLZR *C*. *parapsilosis* isolates overexpressed *CDR1* (Souza et al., 2015). This observation contrasts with FLZR *C*. *albicans* isolates, which are believed not to be horizontally transmitted and show significant genetic diversity. This phenomenon also has implications for mutants lacking either of those transcription factors, and the results from a given background may not be applicable to others, which again warrants further research.

The fact that Brazilian *C*. *parapsilosis* had an extra mutation in *CDR1* may also point to the fact that such isolates may have a higher tendency to acquire mutations. For instance, specific genotypes of *C*. *glabrata* harboring specific mutations of the mismatch repair gene *MSH2* were hypothesized to have a higher rate of mutation frequency (Healey et al., 2016). Whether such a phenomenon is applicable to *C*. *parapsilosis* is yet to be defined and is worth exploring. Given the region-specific diversity and to gain deeper insight into azole resistance in *C*. *parapsilosis*, our study warrants collecting isolates from multiple clinical centers most severely affected by clonal outbreaks, combined with gene expression analysis, sequencing, and application of precise genome editing tools to unravel mechanisms underpinning azole resistance.

Because azole resistance is a growing clinical problem, we sought to determine the efficacy of MOX in combination with fluconazole, and we included FLZR *C*. *parapsilosis* isolates harboring various *ERG11* mutations from both countries (**Table 3**). Our data revealed that MOX *per se* does not have antifungal activity (**Table 3**), which is unlike the observations made for *C. albicans* and *C*. *glabrata* (Silva et al., 2013). Yet, in line with the observation that MOX may have activity beyond efflux pump inhibition (Silva et al., 2013), we observed that when combined with fluconazole, MOX significantly potentiated fluconazole activity by reducing fluconazole MIC 8– to 32–fold (**Table 3**) regardless of the underlying fluconazole resistance mechanisms (*ERG11* mutation alone or in combination with *CDR1* overexpression). Likewise, fluconazole dramatically decreased the MIC of MOX 64– to 128–fold, which mirrors the potent synergistic activities exerted by their combination.

**Table 3:** The minimum inhibitory concentrations of fluconazole and/or micafungin in combination with milbemycin oxim.

Strain #	Erg11	Milb alone	FLZ alone	Milb combination	FLZ combination	FIC index	Activity
**T3**	Y132F	>64	4	0.5	1	0.257813	Synergistic
**T10**	Y132F+G307A	>64	64	1	16	0.265625	Synergistic
**T14**	G458S	>64	64	1	8	0.140625	Synergistic
**B6**	Y132F	>64	16	0.5	2	0.132813	Synergistic
**Strain #**	**Fks1**	**Milb alone**	**MICA alone**	**Milb combination**	**MICA combination**	**FIC index**	**Activity**
T17^*^	R658G	>64	>16	>64	>16	2	Indifference

FIC, Fractional inhibitory concentration; Milb, milbemycin oxim; FLZ, Fluconazole; MICA, Micafungin.

*T17 is a multidrug resistant C. parapsilosis isolate that carries Y132F+K143R in Erg11.

As MDR is an emerging clinical problem in our center in Turkey, we were curious to explore the efficacy of the combination of micafungin and MOX against an MDR isolate. Of note, the MOX and micafungin combination did not yield synergistic or additive activities, and the MICs did not change compared to each drug alone. We note that *in vivo* studies are required to validate the synergistic and indifferent activities observed for fluconazole-MOX and micafungin-MOX, respectively. Of note, given that checkerboard assay is labor-intensive and time-consuming, here we just included a limited number of isolates and the lack of efficacy of MOX and micafungin warrants inclusion of further isolates.

Collectively, our study shows that fluconazole resistance mechanisms employed by *C*. *parapsilosis* isolates can vary from hospital to hospital and among geographical regions and that efflux pump inhibitors such as MOX in combination with fluconazole showed promising activities against FLZR isolates.

## Data availability statement

The original contributions presented in the study are included in the article/**supplementary material**. Further inquiries can be directed to the corresponding authors.

## Ethics statement

This study is part of a FAPESP GRANT (2017/02203-7) that was reviewed and approved by local Ethical Committee at UNIFESP.

## Author contributions

Conceptualization, AA, FD, and DP. Methodology, FD and AA. Software, FD, AA, and ES. Validation, FD and AA. Formal analysis, FD and AA. Investigation, FD and AA. Resources, MI, SH-P, JA, AC, and LF. Data curation, FD and AA. Writing – original draft, FD, AA, and ES. Writing – review and editing, all co-authors. Visualization, FD, AA, and ES. Supervision, DP, FD, and AA. Project administration, FD and AA. Funding acquisition, DP and AC. All authors contributed to the article and approved the submitted version.

## Funding

This work was supported by internal support by the Center for Discovery and Innovation to DP and Grant 2017/02203-7, Fundação de Amparo a Pesquisa de Sao Paulo (FAPESP) to AC.

## Conflict of interest

AC received educational Grants from Angem, Eurofarma, Knight-United Medical, Gilead, Pfizer and travel grant support from Eurofarma and Knight-United Medical.

DP receives research support and/or serves on advisory boards for Amplyx, Cidara, Scynexis, N8 Medical, Merck, Regeneron, and Pfizer. He also has a patent covering the detection of fungal species and drug resistance, as well as a pending patent on COVID-19 detection licensed to T2 Biosystems.

The remaining authors declare that the research was conducted in the absence of any commercial or financial relationships that could be construed as a potential conflict of interest.

## Publisher’s note

All claims expressed in this article are solely those of the authors and do not necessarily represent those of their affiliated organizations, or those of the publisher, the editors and the reviewers. Any product that may be evaluated in this article, or claim that may be made by its manufacturer, is not guaranteed or endorsed by the publisher.

## Supplementary material

Supplementary Figure 1
**(A)** Basal expression of Turkish isolates carrying G458S was the highest, while the rest had the same level of expression. **(B)** Basal overexpression of *CDR1* was noted only for one Brazilian isolate carrying L518F in *TAC1.* **(C)** All of the isolates tested showed the same basal level of *MDR1* expression. The basal expression level was calculated using 2^-ΔCt^, where ΔCt refers to the Ct of the target gene- Ct of *ACT1*.
