# Supplementary figures and images for: Determinants of fluconazole resistance and the efficacy of fluconazole and milbemycin oxim combination against Candida parapsilosis clinical isolates from Brazil and Turkey
Source: Front Fungal Biol. 2022 Jul 28;3:906681. doi: 10.3389/ffunb.2022.906681 (PMC10512262; doi:10.3389/ffunb.2022.906681)

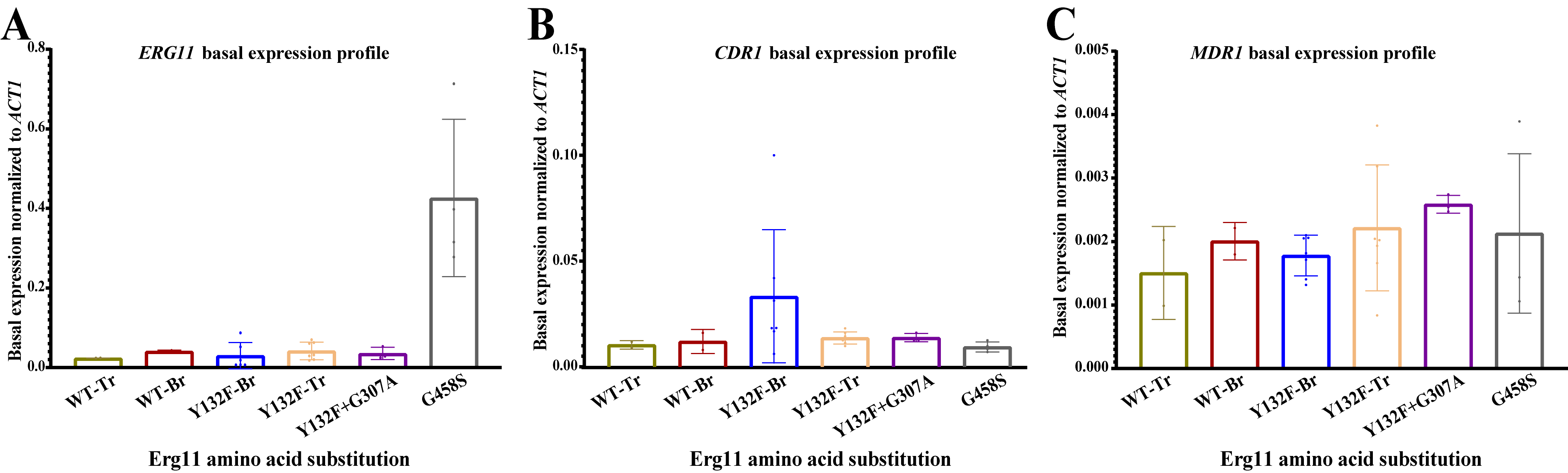

Supplement: Supplementary Figure 1 — (A) Basal expression of Turkish isolates carrying G458S was the highest, while the rest had the same level of expression. (B) Basal overexpression of CDR1 was noted only for one Brazilian isolate carrying L518F in TAC1. (C) All of the isolates tested showed the same basal level of MDR1 expression. The basal expression level was calculated using 2-ΔCt, where ΔCt refers to the Ct of the target gene- Ct of ACT1. [file Image_1.tif]
