# Supplementary material for: Determinants of fluconazole resistance and the efficacy of fluconazole and milbemycin oxim combination against Candida parapsilosis clinical isolates from Brazil and Turkey
Source: Front Fungal Biol. 2022 Jul 28;3:906681. doi: 10.3389/ffunb.2022.906681 (PMC10512262; doi:10.3389/ffunb.2022.906681)
Supplement: Supplementary file 2 [file Table_1.docx]

**Supplementary Table 1.** List of primers and qPCR conditions used to determine the expression levels of *CDR1*, *ERG11*, and *MDR1*.

| **Loci** | **Primer name** | **Primer sequence** | **PCR conditions** | **R^2^ value** | **Efficiency** | **Melting temperature** |
| --- | --- | --- | --- | --- | --- | --- |
| ***Actin*** | **ACT1-F** | 3^'^-AGAATCGATTTGGCTGGTAG-5^'^ | 42°C, 5 mins, 95°C, 10 secs, 40 cycles of (95°C, 5 secs, 60°C, 30 secs), melting temperature determination (95°C, 1 min, 60°C, 30 secs, 95°C, 30 secs) | ≥99% | 98% | 81.2^°^C±0.45 |
|  | **ACT1-R** | 3^'^-CACAATTTCTCCTTGATGTCTC-5^'^ |  |  |  |  |
| ***CDR1*** | **CDR1-F** | 3^'^-GCACATGTTCAGATCCATTG-5^'^ | 42°C, 5 mins, 95°C, 10 secs, 40 cycles of (95°C, 5 secs, 60°C, 30 secs), melting temperature determination (95°C, 1 min, 60°C, 30 secs, 95°C, 30 secs) | ≥99% | 98% | 83.65^°^C±0.36 |
|  | **CDR1-R** | 3^'^-GATCCATCTAGACCATCCCA-5^'^ |  |  |  |  |
| ***ERG11*** | **ERG11-F** | 3^'^-GTTGGTTCAGCCGTATCTT-5^'^ | 42°C, 5 mins, 95°C, 10 secs, 40 cycles of (95°C, 5 secs, 60°C, 30 secs), melting temperature determination (95°C, 1min, 60°C, 30 secs, 95°C, 30 secs) | ≥99% | 96% | 78.8^°^C±0.52 |
|  | **ERG11-R** | 3^'^-CACCGTCATTACTCTACCCA-5^'^ |  |  |  |  |
| ***MDR1*** | **MDR1-F** | 3^'^-GACAACATTGCTGGGTTTTG-5^'^ | 42°C, 5 mins, 95°C, 10 secs, 40 cycles of (95°C, 5 secs, 60°C, 30 secs), melting temperature determination (95°C, 1 min, 60°C, 30 secs, 95°C, 30 secs) | ≥99% | 95% | 83.68^°^C±0.43 |
|  | **MDR1-R** | 3^'^-TCCATAATGCCAACGACAAG-5^'^ |  |  |  |  |
